# Supplementary material for: Endometrial PTEN Deficiency Leads to SMAD2/3 Nuclear Translocation
Source: Cancers (Basel). 2021 Oct 5;13(19):4990. doi: 10.3390/cancers13194990 (PMC8507901; doi:10.3390/cancers13194990)
Supplement: Supplementary file 1 [file cancers-13-04990-s001.zip › cancers-1327119-supplementary.pdf]

Supplementary Material: Endometrial PTEN Deficiency Leads to Tumor Suppressive SMAD2/3 Nuclear Translocation

Nuria Eritja, Raul Navaridas, Anna Ruiz-Mitjana, Maria Vidal-Sabanes, Joaquim Egea, Mario Encinas, Xavier Matias-Guiu and Xavi Dolcet

A

Cre:ER+/-;Pten fl/fl	Relative fold increase of Phospo-SMAD2/3 (Total SMAD2/3)	SEM	p-value
NO TAM	1.0011	0.1605	0.956
TAM	1.0454	0.07	

Cre:ER+/-;Pten fl/fl	Relative fold increase of Total SMAD2/3 (Tubulin)	SEM	p-value
NO TAM	1.0001	0.0992	* 0.027
TAM	2.575	0.04625	

Cre:ER+/-;Pten fl/fl	Relative fold increase of Phospo-SMAD2/3 (Tubulin)	SEM	p-value
NO TAM	0.99950	0.0992	** 0.009
TAM	2.7425	0.04625	

T-test (Mann-Whitney test)

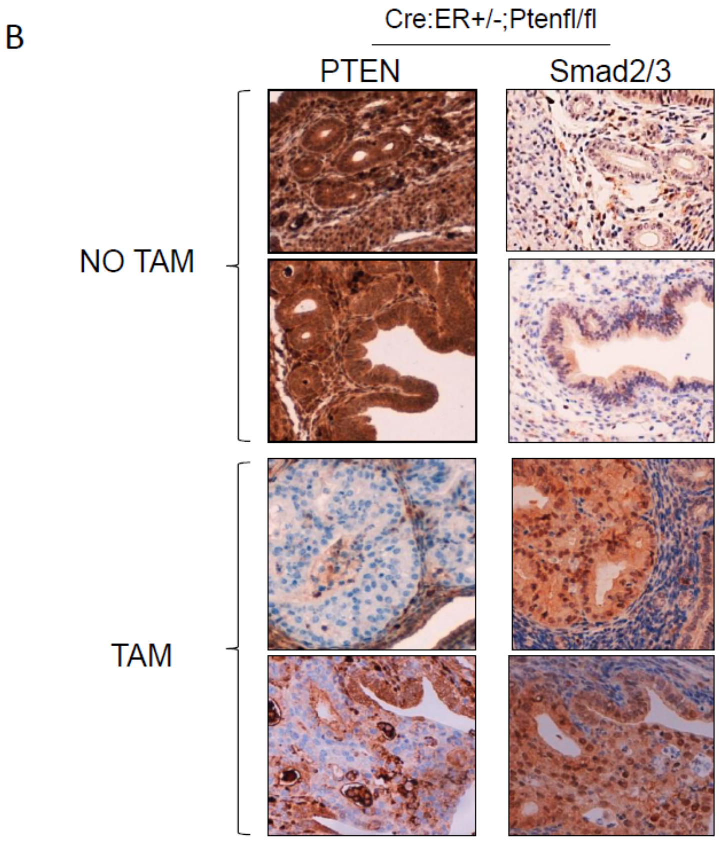

Figure S1. PTEN deletion induces Smad2/3 expression and nuclear translocation of Smad2/3 in vivo. **(A)** Densitometry quantification and statistical analysis corresponding to western blot shown in Figure 5A Values are mean ± s.e.m. $* \, p < 0.05$ and $** \, p < 0.01$, by t-test analysis. **(B)** PTEN and Smad2/3

immunohistochemistry on formalin-fixed paraffin-embedded endometrial tissue sections from 12-week-old Cre:ER+/-; Pten fl/fl mice that have been injected (TAM) or not (NO TAM) with tamoxifen to induce PTEN deletion (4 weeks).

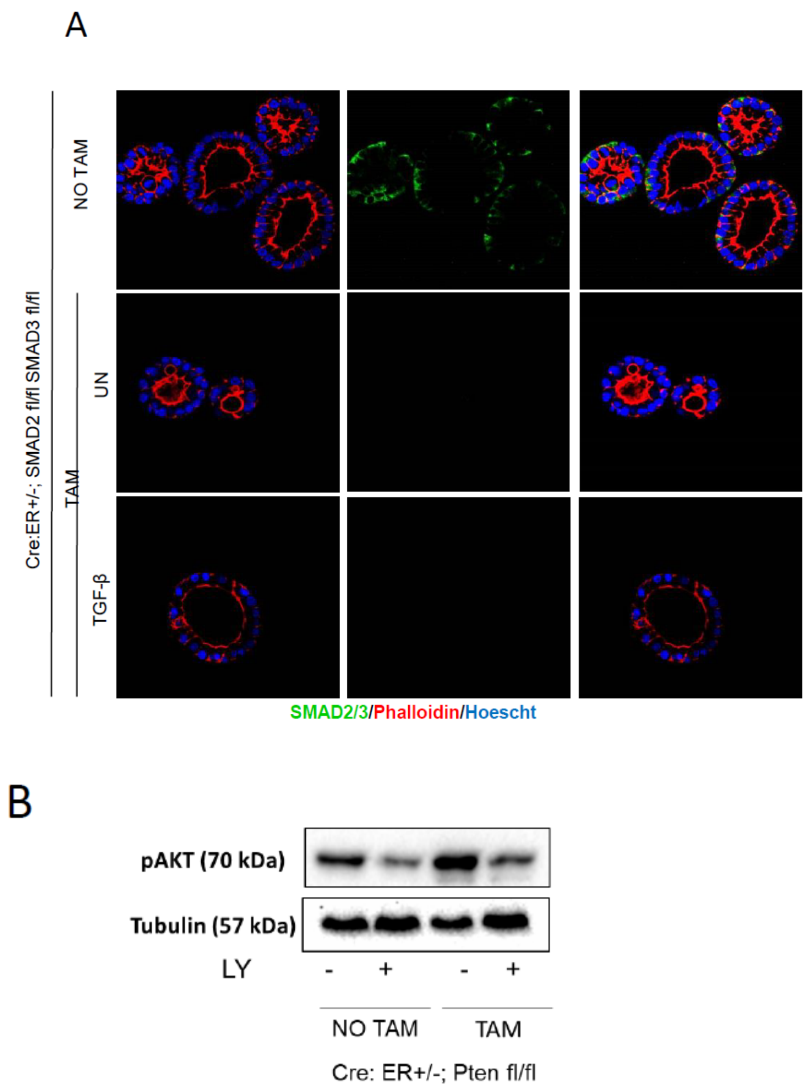

Figure S2. (A) Deletion of SMAD2/3 demonstrates the specificity of SMAD2/3 immunofluorescence. SMAD2/3 Immunofluorescence of organoid cultures from Cre:ER+/-; SMAD2fl/flSMAD3fl/fl mice treated or not with Tamoxifen (TAM) to induce deletion of floxed alleles. Tamoxifen treated cultures were also stimulated with 10ng/ml of TGF-β for 1 h or left untreated (UN). Cells were also stained with phalloidin to evidence actin cytoskeleton and glandular morphology. Nuclei were counter-stained with Hoechst. **(B)** Western blot analysis of p-AKT on lysates from Cre:ER+/-; Ptenfl/fl organoid cultures treated (TAM) or not (NOTAM) with tamoxifen and treated with (+) or without (-) 10μM LY294002(LY).

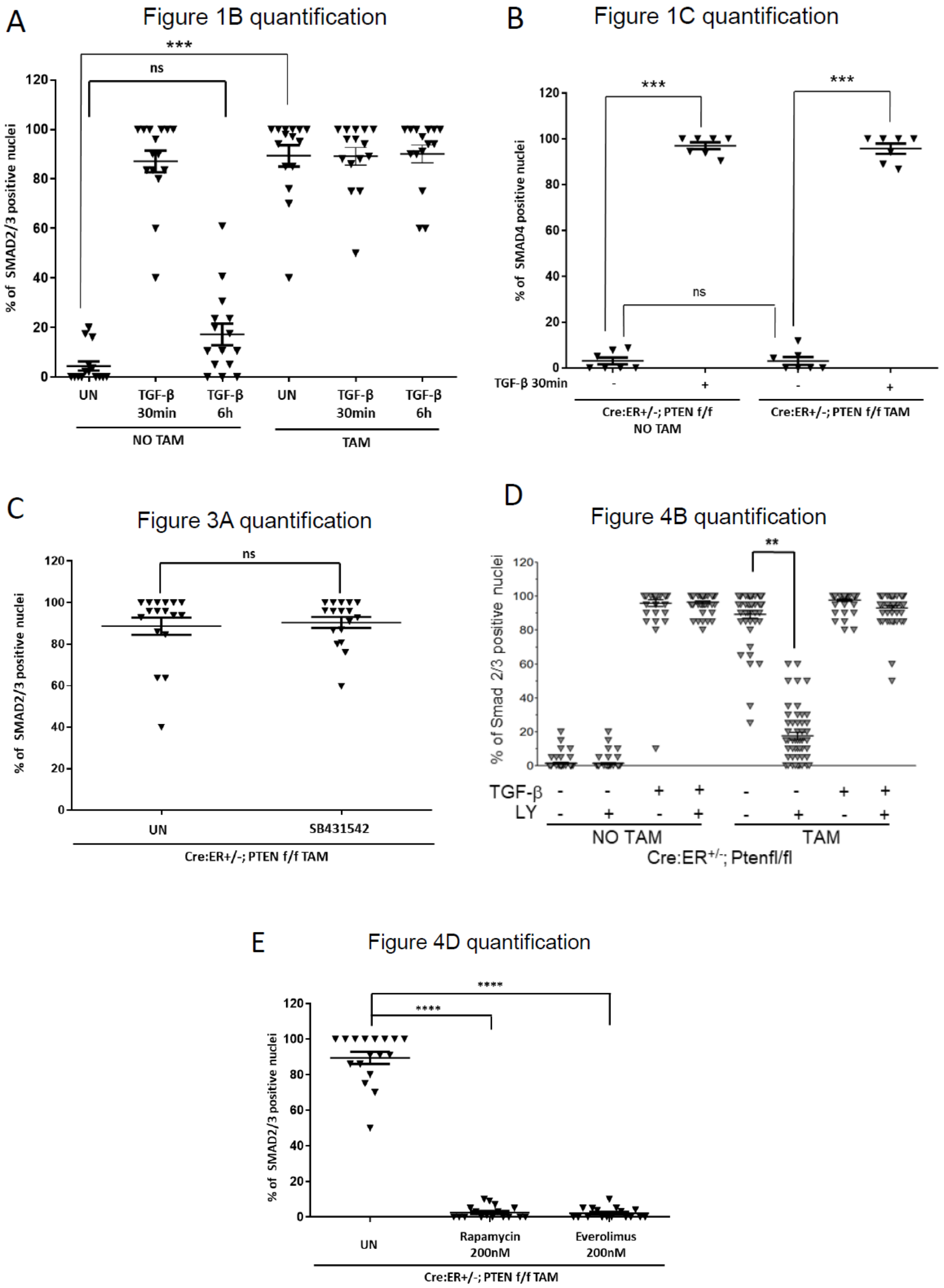

Figure S3: Quantification of nuclear localization of SMAD2/3 or SMAD4 corresponding to the indicated Figures. Experiments were performed three independent times (each with n = 30 total cells per gland counted per condition). Asterisks denote statistically significant differences between conditions. *** $p \le 0.01$ (one-way ANOVA Tukey's multiple comparisontest).

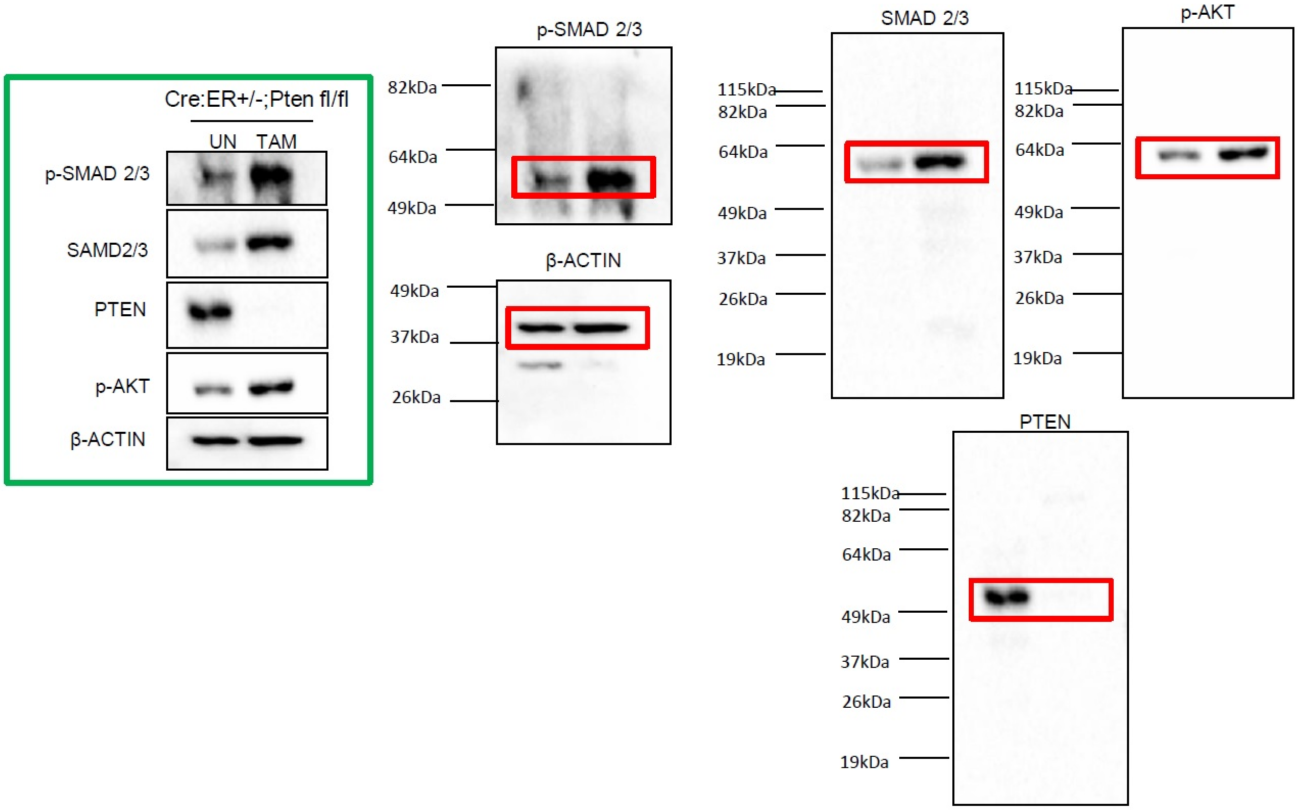

Figure S4. Uncropped Figure 1A.

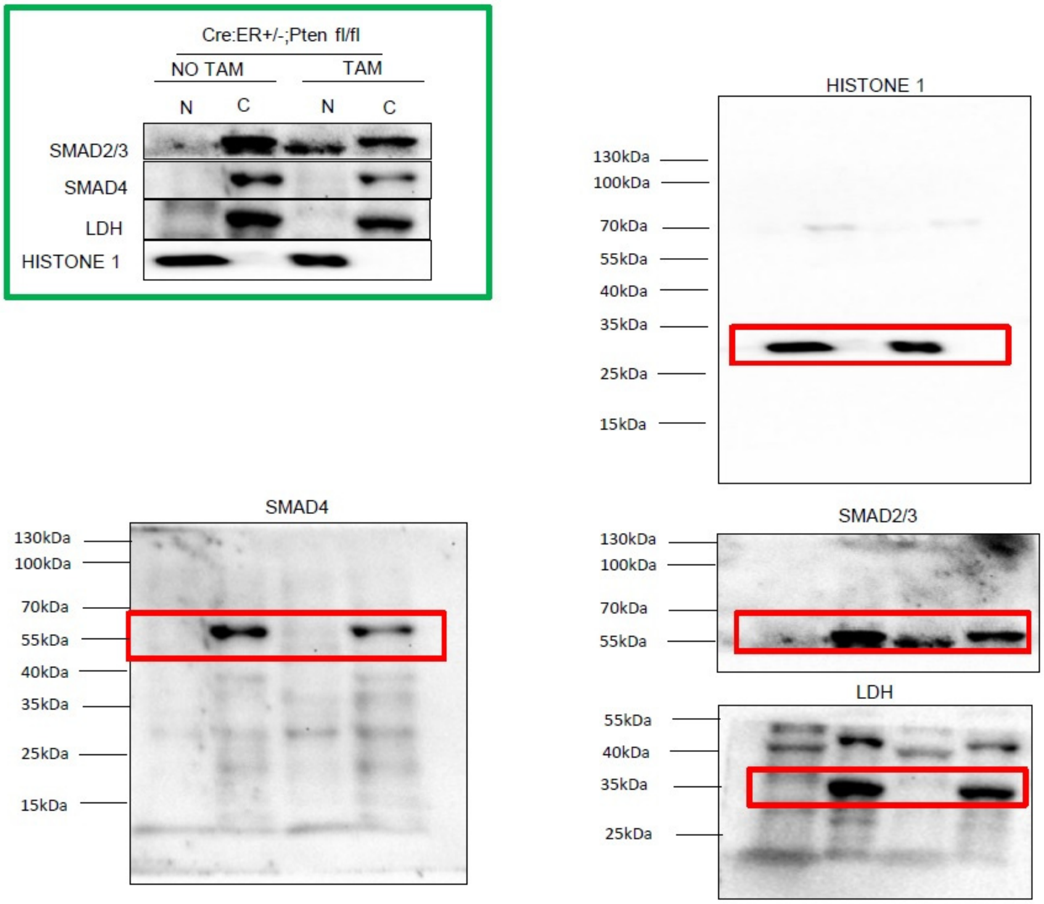

Figure S5. Uncropped Figure 1D.
